# Crystal structure of *catena*-poly[[[bis­(1-benzyl­imidazole-κ*N*)copper(II)]-μ-sulfato-κ^2^ *O*:*O*′-[tetra­kis­(1-benzyl­imidazole-κ*N*)copper(II)]-μ-sulfato-κ^2^ *O*:*O*′] *N*,*N*-di­methyl­formamide disolvate dihydrate]

**DOI:** 10.1107/S2056989022008714

**Published:** 2022-09-06

**Authors:** Nareekarn Meebua, Wanatchaporn Pentes, Kittipong Chainok, Sakchai Laksee, Nanthawat Wannarit

**Affiliations:** a Department of Chemistry, Faculty of Science and Technology, Thammasat University, Klong Luang, Pathum Thani 12121, Thailand; b Thammasat University Research Unit in Multifunctional Crystalline Materials and Applications (TU-MCMA), Faculty of Science and Technology, Thammasat University, Klong Luang, Pathum Thani 12121, Thailand; c Nuclear Technology Research and Development Center, Thailand Institute of Nuclear Technology (Public Organization), Nakhon Nayok 26120, Thailand; Vienna University of Technology, Austria

**Keywords:** crystal structure, copper(II), 1-benz­yl­imidazole, bridging sulfate ligand, chain structure

## Abstract

The title one-dimensional copper(II) coordination polymer, {[Cu(SO_4_)(C_10_H_10_N_2_)_3_]·C_3_H_7_NO·H_2_O}_*n*_ or {[Cu(bzi)_3_(μ-O_2_SO_2_)]·H_2_O·DMF}_*n*_ (bzi = 1-benz­yl­imidazole, C_10_H_10_N_2_; DMF = *N*,*N*-di­methyl­formamide, C_3_H_7_NO), is constructed by monodentate bzi ligands and bridging sulfate anions, leading to chains propagating parallel to the *c* axis. Within a chain, there are two crystallographic independent Cu^II^ ions, each with site symmetry  , which form [CuN_2_O_2_] and [CuN_4_O_2_] polyhedra alternating along the chain direction. The crystal structure is consolidated by weak hydrogen-bonding, C—H⋯π and π–π inter­actions, leading to the formation of a three-dimensional supra­molecular network.

## Abstract 2

The crystal structure of the title compound comprises polymeric chains formed by two independent copper(II) polyhedra, [CuN_2_O_2_] and [CuN_4_O_2_], which are linked by sulfate anions and stabilized by extended hydrogen-bonding, C—H⋯π and π–π inter­actions.

## 1. Chemical context

The exploration of new transition-metal coordination polymers (CPs) is still an ongoing process since this class of mol­ecular materials presents inter­esting properties and potential applications in adsorption, catalysis, storage, and photoluminescent sensing (Engel & Scott, 2020 ▸; Liu *et al.*, 2021 ▸; Baruah, 2022 ▸; Ma & Horike, 2022 ▸). For the design and synthesis of new CPs, metal ions and bridging ligands play an important role, because they influence structural topologies, dimensionalities, and possible functions (Du *et al.*, 2013 ▸). In this context, we focused on the copper(II) ion and *O*-donor sulfate (SO_4_^2–^) and *N*-donor heterocyclic aromatic ligands for the current study. Copper(II) compounds show inter­esting electronic and magnetic properties, accompanied by various structural topologies, physical properties and applications (Das & Pal, 2001 ▸; Gao & Liu, 2022 ▸). The sulfate anion can act as a bridging ligand due to its versatile coordination modes supporting the increase of structural dimensionalities of the CPs (Yotnoi *et al.*, 2014 ▸). The presence of mono- and/or bidentate *N*-donor heterocyclic aromatic imidazole derivatives as ligands in CPs is generally found to increase the extended structures and the stability of the crystal structures through supra­molecular inter­actions such as π–π stacking and C—H⋯π bonding (Krinchampa *et al.*, 2016 ▸; Assavajamroon *et al.*, 2019 ▸). As previous studies suggest, there is limited research reported for Cu^II^ CPs constructed from mixed sulfate and *N*-donor imidazole derivatives, for example [Cu(*L*)_2_(μ-O_2_SO_2_)]_*n*_ where *L* = imidazole (Fransson & Lundberg, 1972 ▸; Kumar *et al.*, 2014 ▸) and *L* = *N*-methyl­imidazole (Liu *et al.*, 2003 ▸). During the current study, we used the imidazole derivative, 1-benzyl­imidazole (bzi), to investigate its influence on supra­molecular inter­actions in the resulting network. 
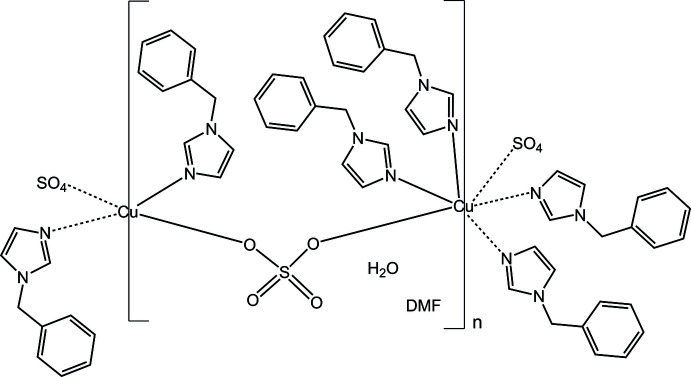


In the present communication, we report the crystal structure, spectroscopic characteristics and some physical properties of {[Cu(bzi)_3_(μ-O_2_SO_2_)]·H_2_O·DMF}_n_ (bzi = 1-benz­ylimidazole; DMF = *N*,*N*-di­methyl­formamide).

## 2. Structural commentary

**Figure 1:**
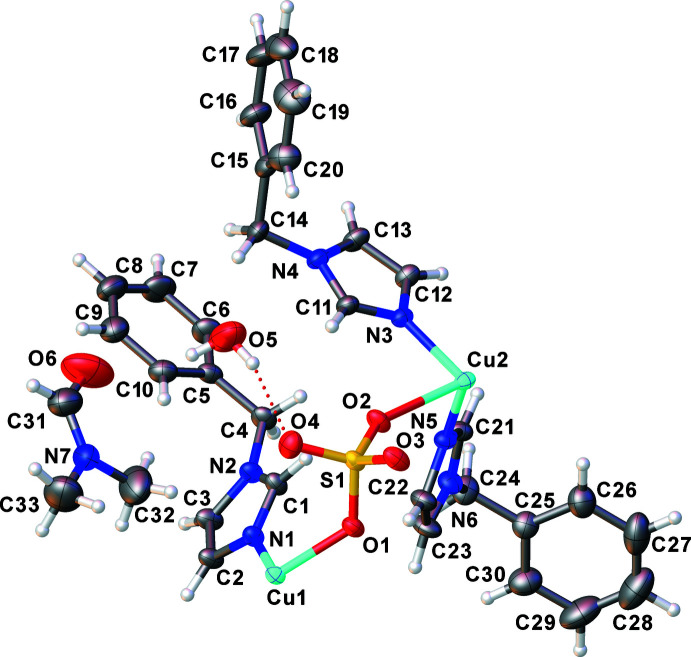
Asymmetric unit of the title compound with displacement ellipsoids drawn at the 30% probability level.

**Figure 2:**
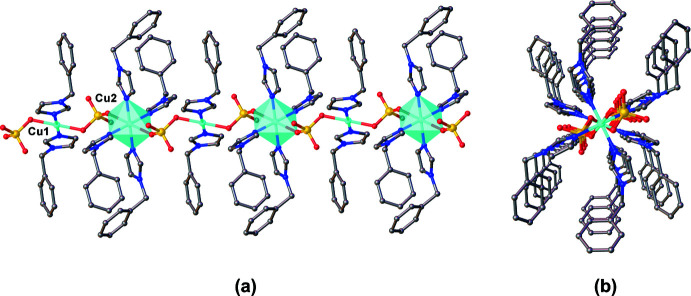
Side (*a*) and top (*b*) views of the chain-like structure of the title compound extending parallel to the *c* axis. Hydrogen atoms bound to carbon atoms as well as solvent water and DMF mol­ecules were omitted for clarity.

The asymmetric unit of the solvated coordination polymer {[Cu(bzi)_3_(μ-O_2_SO_2_)]·H_2_O·DMF}_n_ comprises two Cu^II^ ions with site symmetry 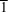 (Wyckoff letters *b* and *d*), three bzi mol­ecules (see Fig. S1 in the supporting information), a coordinating sulfate anion, one water and one DMF solvent mol­ecule (Fig. 1 ▸). The environments of the two Cu^II^ cations are different. Cu1 is surrounded by two nitro­gen donor atoms from two monodentate bzi ligands and two oxygen donor atoms of two different sulfate bridging ligands, resulting in an [N_2_O_2_] coordination set with a square-planar shape and Cu1—N1 and Cu1—O1 bond lengths of 1.9951 (14) and 1.9564 (12) Å, respectively; the bite angles around Cu1 are in the range 89.25 (6)–90.75 (6)°. Cu2 is coordinated by four nitro­gen donor atoms from four monodentate bzi ligands and two oxygen donor atoms of two different sulfate bridging ligands, resulting in an [N_4_O_2_] coordination set with a typically Jahn–Teller-distorted octa­hedral shape with bond lengths of Cu2—N3 = 2.0210 (15), Cu2—N5 = 2.013 (15) Å, and Cu2—O2 = 2.4912 (12) Å. Both Cu^II^ sites are alternatively connected by bis-monodentately binding and bridging sulfate ligands, μ-κ^2^ *O*,*O*′, leading to a chain-like structure extending parallel to the *c* axis, as shown in Fig. 2 ▸. The Cu1⋯Cu2 distance within a chain is 6.1119 (4) Å.

## 3. Supra­molecular features

**Figure 3:**
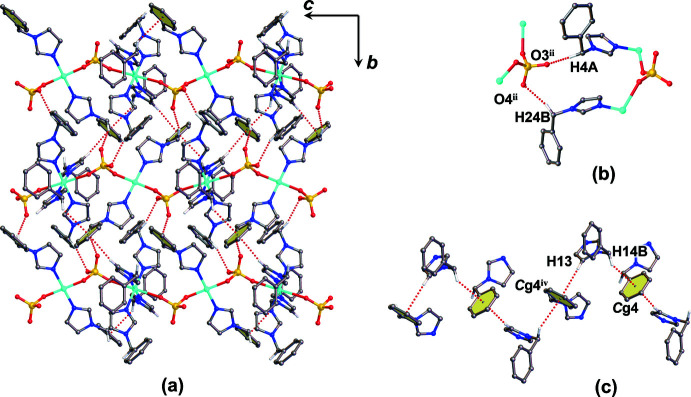
(*a*) View of the two-dimensional supra­molecular network of the title compound formed through (*b*) hydrogen bonding-inter­actions between methyl­ene groups and the sulfate ligand, and (*c*) C—H⋯π inter­actions between adjacent chains. [Symmetry codes: (ii) −*x* + 1, *y* − 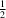 , −*z* + 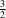 ; (iv) *x*, −*y* + 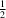 , *z* − 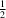 .]

**Figure 4:**
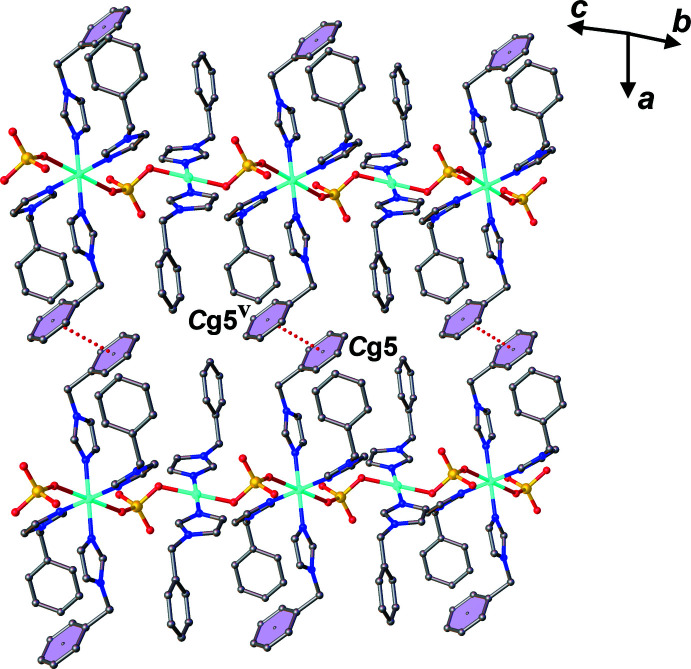
View of inter­chain π–π inter­actions in the title compound along the *a* axis. [Symmetry code: (v) −*x*, −*y* + 1, −*z* + 1].

**Figure 5:**
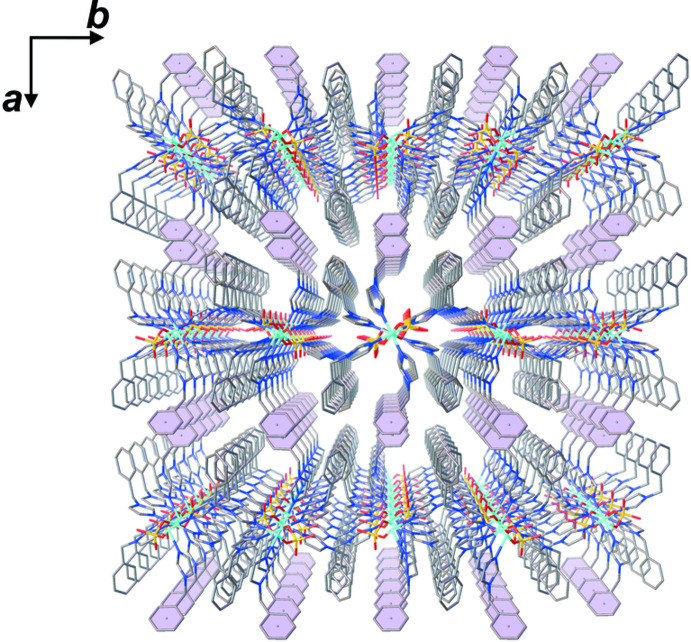
View of the three-dimensional supra­molecular network of the title compound. Solvent water and DMF mol­ecules are omitted for clarity.

The crystal structure of the title compound is consolidated by weak inter­actions such as hydrogen-bonding, C—H⋯π and π–π inter­actions. Non-classical C—H⋯O hydrogen-bonding inter­actions are found between the C—H donor groups of the bzi imidazole rings to three different oxygen acceptor atoms (O1, O2 and O3) of a sulfate bridging ligand, together with a weak hydrogen bond within a bzi mol­ecule, C10—H10⋯N2 (Table 1 ▸; Fig. S2 in the supporting information). Moreover, O—H⋯O hydrogen-bonding inter­actions between the bridging sulfate ion in the chain and the solvate water and DMF mol­ecules are found (Table 1 ▸; Fig. S3 in the supporting information). Inter­molecular inter­actions between adjacent chains (Fig. 3 ▸ *a*) exist through hydrogen-bonding inter­actions between a methyl­ene group and a sulfate ligand, C4—H4⋯O3^ii^ and C24—H24*B*⋯O4^ii^ (Table 1 ▸; Fig. 3 ▸ *b*) and by C—H⋯π inter­actions, C13—H13⋯*Cg*4^iv^ and C14—H14*B*⋯*Cg*4^iv^ (Table 1 ▸; Fig. 3 ▸ *c*), leading to a two-dimensional supra­molecular network extending parallel to the *bc* plane, as shown in Figs. S3 and S4 in the supporting information. Furthermore, π–π stacking inter­actions are present between the phenyl rings of bzi ligands (Fig. 4 ▸) with a centroid-to-centroid distance *Cg*5⋯*Cg*5^v^ of 3.7099 (18) Å along the *a-*axis direction [*Cg*5 is the centroid of the C15–C20 phenyl ring; symmetry code: (v) −*x*, −*y* + 1, −*z* + 1], eventually leading to a three-dimensional supra­molecular framework of the title compound, as shown in Fig. 5 ▸.

## 4. Spectroscopic characterization

The FT–IR spectrum of the title compound (Fig. S5 in the supporting information) exhibits the characteristic broad bands (centered at 3454 cm^−1^) assigned to the O—H stretching vibration of the solvent water mol­ecule hydrogen-bonded to the DMF solvent mol­ecule. Characteristic bands of the bzi ligand are observed at 3142 cm^−1^ for the aromatic C—H stretching, at 1523 and 1453 cm^−1^ and in the range of 700–500 cm^−1^ for the C=C, C—N stretching and C—H bending, respectively (Assavajamroon *et al.*, 2019 ▸). The strong bands at 1675, 1116 and 713 cm^−1^ are due to the asymmetric stretching of the bridging sulfate ligand (Wang *et al.*, 2014 ▸).

The solid-state diffuse reflectance spectrum of the title compound (Fig. S6 in the supporting information) shows a broad asymmetric band with λ_max_ at 602 nm (16.60 kK) and a shoulder at about 756 nm (13.24 kK). These bands might be assigned to electronic *d* → *d* transitions, (*d*_*xy*_, *d*_*xz*_, *d*_*yz*_) → *d*_*x* ^2^__–y^2^_ and *d*_*z* ^2^_→ *d*_*x* ^2^__–*y* ^2^_, corresponding to a distorted octa­hedral conformation.

## 5. PXRD and thermal analysis

The plots of the experimental and simulated powder X-ray diffraction (PXRD) patterns of the title compound (Fig. S7 in the supporting information) show a good match, confirming reproducibility and phase purity.

The thermal stability of the title compound has been investigated by means of thermogravimetric analysis with the temperature in the range 303–1073 K under a nitro­gen atmosphere. Based on the results (Fig. S8 in the supporting information), the title compound is stable to about 371 K. Above this temperature, the compound starts to decompose by a mass loss of 13%, which corresponds to the loss of solvent water and DMF mol­ecules. The second step of mass loss (65%) corresponds to the release of the remaining coordinating bzi and sulfate ligands. Further increasing the temperature leads to another mass loss (22%) until CuO forms as the final product.

## 6. Database survey

According to a search of the Cambridge Structural Database (CSD; version 5.41, November 2019 update; Groom *et al.*, 2016 ▸), there are some one-dimensional Cu^II^ coordination polymers containing the sulfate anion as a bridging ligand together with *N*-donor imidazole-based ligands. The ones most closely related to the title compound are [Cu(imida­zole)_4_SO_4_] (TIMZCU02; Kumar *et al.*, 2014 ▸) and [Cu(*N*-methyl­imidazole)_4_(SO_4_)] (IJEBII; Liu *et al.*, 2003 ▸). These two Cu^II^ coordination polymers have the same octa­hedral [N_4_O_2_] coordination set around the Cu^II^ ion, while those of the title compound contain alternatively two different Cu^II^ polyhedra, as discussed in the *Structural commentary*.

## 7. Synthesis and crystallization

A methano­lic solution (5 ml) of bzi (0.6329 g, 4.0 mmol) was dropped slowly into a methano­lic solution (5 ml) of CuSO_4_·5H_2_O (0.2491 g, 1.0 mmol) under continuous stirring at 333 K over a period of 10 min, resulting in a blue solution. The solution was then filtered and allowed to evaporate slowly under atmospheric conditions at room temperature. After seven days, the solution became viscous, and 10 ml of DMF were added to the solution under continuous stirring at 333 K over a period of 5 min. Stirring was continued until the solution became clear. Finally, the solution was filtered and allowed to evaporate slowly in air at room temperature. Blue crystals of the title compound were obtained within a day (yield 38%, 93.4 mg, based on the Cu^II^ salt).

## 8. Refinement

Crystal data, data collection and structure refinement details are summarized in Table 2 ▸. All C-bound H atoms were calculated and refined using a riding model, with C—H = 0.93 Å for aromatic H atoms (0.97 Å for methyl H atoms), and *U*_iso_(H) = 1.2*U*_eq_(C) [*U*_iso_(H) = 1.5*U*_eq_(C)]. The O-bound H atoms of the water mol­ecule were located in a difference-Fourier map, and were refined with an O—H bond length of 0.85 Å, and with *U*_iso_(H) = 1.5*U*_eq_(O).

## Supplementary Material

Crystal structure: contains datablock(s) I. DOI: 10.1107/S2056989022008714/wm5658sup1.cif

Structure factors: contains datablock(s) I. DOI: 10.1107/S2056989022008714/wm5658Isup2.hkl

The supporting information about crystal structures and characterization has been presented in the pdf file. DOI: 10.1107/S2056989022008714/wm5658sup3.pdf

CCDC reference: 2204358

Additional supporting information:  crystallographic information; 3D view; checkCIF report

## Figures and Tables

**Table 1:** Hydrogen-bond geometry (Å, °) *Cg* is the centroid of the C5–C10 ring.

*D*—H⋯*A*	*D*—H	H⋯*A*	*D*⋯*A*	*D*—H⋯*A*
O5—H5*A*⋯O4	0.85	1.96	2.772 (3)	159
O5—H5*B*⋯O6	0.85	2.29	3.106 (5)	160
C1—H1⋯O2	0.93	2.23	3.131 (2)	164
C2—H2⋯O3^i^	0.93	2.34	3.227 (2)	159
C4—H4*A*⋯O3^ii^	0.97	2.55	3.445 (3)	153
C10—H10⋯N2	0.93	2.54	2.866 (3)	101
C11—H11⋯O2	0.93	2.40	2.990 (2)	121
C12—H12⋯O3^iii^	0.93	2.48	3.378 (2)	162
C14—H14*A*⋯O5	0.97	2.50	3.425 (4)	159
C21—H21⋯O2^iii^	0.93	2.57	3.038 (2)	112
C21—H21⋯O3^iii^	0.93	2.36	3.274 (2)	170
C24—H24*B*⋯O4^ii^	0.97	2.40	3.346 (3)	164
C32—H32*A*⋯O6	0.96	2.29	2.705 (7)	105
C13—H13⋯*Cg*4^iv^	0.93	2.87	3.759 (3)	161
C14—H14*B*⋯*Cg*4^iv^	0.97	2.99	3.711 (3)	132

Symmetry codes: (i) 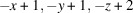 ; (ii) 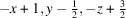 ; (iii) 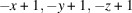 ; (iv) 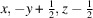 .

**Table 2:** Experimental details

Crystal data	
Chemical formula	[Cu(SO_4_)(C_10_H_10_N_2_)_3_]·C_3_H_7_NO·H_2_O
*M*_r_	725.31
Crystal system, space group	Monoclinic, *P*2_1_/*c*
Temperature (K)	296
*a*, *b*, *c* (Å)	15.8896 (10), 18.1195 (11), 12.2238 (7)
β (°)	94.239 (2)
*V* (Å^3^)	3509.7 (4)
*Z*	4
Radiation type	Mo *K*α
μ (mm^−1^)	0.74
Crystal size (mm)	0.38 × 0.35 × 0.32

Data collection	
Diffractometer	Bruker D8 QUEST CMOS PHOTON II
Absorption correction	Multi-scan (*SADABS*; Krause *et al.*, 2015 ▸).
*T*_min_, *T*_max_	0.640, 0.746
No. of measured, independent and observed [*I* > 2σ(*I*)] reflections	50998, 10697, 7310
*R*_int_	0.054
(sin θ/λ)_max_ (Å^−1^)	0.714

Refinement	
*R*[*F* ^2^ > 2σ(*F* ^2^)], *wR*(*F* ^2^), *S*	0.044, 0.117, 1.02
No. of reflections	10697
No. of parameters	441
H-atom treatment	H-atom parameters constrained
Δρ_max_, Δρ_min_ (e Å^−3^)	0.39, −0.35

Computer programs: *APEX3* and *SAINT* (Bruker, 2016 ▸), *SHELXT* (Sheldrick, 2015*a* ▸), *SHELXL* (Sheldrick, 2015*b* ▸), *OLEX2* (Dolomanov *et al.*, 2009 ▸) and *publCIF* (Westrip, 2010 ▸).

## supplementary crystallographic information

### Crystal data

**Table:**

[Cu(SO_4_)(C_10_H_10_N_2_)_3_]·C_3_H_7_NO·H_2_O	*F*(000) = 1516
*M**_r_* = 725.31	*D*_x_ = 1.373 Mg m^−^^3^
Monoclinic, *P*2_1_/*c*	Mo *K*α radiation, λ = 0.71073 Å
*a* = 15.8896 (10) Å	Cell parameters from 9903 reflections
*b* = 18.1195 (11) Å	θ = 2.3–29.9°
*c* = 12.2238 (7) Å	µ = 0.74 mm^−^^1^
β = 94.239 (2)°	*T* = 296 K
*V* = 3509.7 (4) Å^3^	Block, dark blue
*Z* = 4	0.38 × 0.35 × 0.32 mm

### Data collection

**Table:**

BRUKER D8 QUEST CMOS PHOTON II diffractometer	10697 independent reflections
Radiation source: sealed x-ray tube, Mo	7310 reflections with *I* > 2σ(*I*)
Graphite monochromator	*R*_int_ = 0.054
Detector resolution: 7.39 pixels mm^-1^	θ_max_ = 30.5°, θ_min_ = 2.3°
ω and φ scans	*h* = −22→22
Absorption correction: multi-scan (*SADABS*; Krause *et al.*, 2015).	*k* = −25→21
*T*_min_ = 0.640, *T*_max_ = 0.746	*l* = −14→17
50998 measured reflections	

### Refinement

**Table:**

Refinement on *F*^2^	Primary atom site location: dual
Least-squares matrix: full	Hydrogen site location: mixed
*R*[*F*^2^ > 2σ(*F*^2^)] = 0.044	H-atom parameters constrained
*wR*(*F*^2^) = 0.117	*w* = 1/[σ^2^(*F*_o_^2^) + (0.049*P*)^2^ + 1.1168*P*] where *P* = (*F*_o_^2^ + 2*F*_c_^2^)/3
*S* = 1.02	(Δ/σ)_max_ = 0.001
10697 reflections	Δρ_max_ = 0.39 e Å^−^^3^
441 parameters	Δρ_min_ = −0.35 e Å^−^^3^
0 restraints	

### Special details

**Table:**

Geometry. All esds (except the esd in the dihedral angle between two l.s. planes) are estimated using the full covariance matrix. The cell esds are taken into account individually in the estimation of esds in distances, angles and torsion angles; correlations between esds in cell parameters are only used when they are defined by crystal symmetry. An approximate (isotropic) treatment of cell esds is used for estimating esds involving l.s. planes.

### Fractional atomic coordinates and isotropic or equivalent isotropic displacement parameters (Å^2^)

**Table:**

	*x*	*y*	*z*	*U*_iso_*/*U*_eq_	
Cu1	0.500000	0.500000	1.000000	0.02929 (8)	
Cu2	0.500000	0.500000	0.500000	0.03313 (8)	
S1	0.45600 (3)	0.57757 (2)	0.78307 (3)	0.03415 (10)	
O1	0.52363 (8)	0.53965 (7)	0.85659 (10)	0.0390 (3)	
O2	0.44811 (9)	0.53635 (7)	0.67969 (10)	0.0422 (3)	
O3	0.48237 (10)	0.65349 (8)	0.76450 (11)	0.0478 (3)	
O4	0.37859 (10)	0.57424 (9)	0.83974 (13)	0.0548 (4)	
N1	0.45657 (10)	0.40654 (8)	0.93045 (12)	0.0348 (3)	
N2	0.40670 (10)	0.32616 (8)	0.80834 (12)	0.0371 (3)	
N3	0.38640 (9)	0.45109 (8)	0.46926 (12)	0.0360 (3)	
N4	0.25298 (9)	0.42410 (10)	0.48218 (13)	0.0421 (4)	
N5	0.55242 (10)	0.40576 (8)	0.55904 (12)	0.0360 (3)	
N6	0.61544 (11)	0.29797 (9)	0.55906 (14)	0.0452 (4)	
C1	0.43083 (13)	0.39607 (10)	0.82595 (14)	0.0386 (4)	
H1	0.429704	0.432455	0.772195	0.046*	
C2	0.44734 (13)	0.33958 (10)	0.98116 (16)	0.0423 (4)	
H2	0.460152	0.330123	1.055305	0.051*	
C3	0.41682 (14)	0.28984 (11)	0.90649 (16)	0.0461 (5)	
H3	0.404953	0.240457	0.919213	0.055*	
C4	0.37274 (13)	0.29476 (12)	0.70396 (15)	0.0448 (5)	
H4A	0.403354	0.249878	0.689678	0.054*	
H4B	0.381868	0.329382	0.645483	0.054*	
C5	0.28021 (14)	0.27738 (11)	0.70233 (16)	0.0431 (4)	
C6	0.24550 (18)	0.22769 (15)	0.6250 (2)	0.0642 (7)	
H6	0.279780	0.205532	0.575961	0.077*	
C7	0.1604 (2)	0.21093 (19)	0.6203 (3)	0.0835 (9)	
H7	0.137660	0.178043	0.567717	0.100*	
C8	0.1095 (2)	0.24268 (19)	0.6930 (3)	0.0829 (9)	
H8	0.052397	0.230936	0.690094	0.099*	
C9	0.14248 (17)	0.29133 (18)	0.7694 (2)	0.0735 (8)	
H9	0.107963	0.312823	0.818691	0.088*	
C10	0.22742 (15)	0.30879 (13)	0.77361 (19)	0.0560 (6)	
H10	0.249295	0.342443	0.825678	0.067*	
C11	0.31994 (12)	0.46172 (12)	0.52597 (16)	0.0418 (4)	
H11	0.319568	0.491353	0.588053	0.050*	
C12	0.35995 (12)	0.40518 (11)	0.38466 (17)	0.0433 (4)	
H12	0.393428	0.388213	0.330678	0.052*	
C13	0.27752 (13)	0.38826 (12)	0.39170 (19)	0.0497 (5)	
H13	0.244297	0.358230	0.344333	0.060*	
C14	0.16745 (13)	0.42591 (15)	0.52061 (19)	0.0547 (6)	
H14A	0.169174	0.449836	0.591730	0.066*	
H14B	0.147524	0.375799	0.529264	0.066*	
C15	0.10724 (13)	0.46645 (15)	0.44190 (18)	0.0512 (5)	
C16	0.05359 (16)	0.42953 (19)	0.3673 (2)	0.0764 (8)	
H16	0.054466	0.378218	0.366189	0.092*	
C17	−0.00141 (19)	0.4666 (3)	0.2943 (3)	0.0967 (11)	
H17	−0.036817	0.440720	0.243896	0.116*	
C18	−0.0031 (2)	0.5408 (3)	0.2970 (3)	0.0937 (11)	
H18	−0.040304	0.566072	0.248004	0.112*	
C19	0.0481 (2)	0.5794 (2)	0.3696 (3)	0.0999 (11)	
H19	0.045884	0.630676	0.370829	0.120*	
C20	0.1044 (2)	0.54141 (18)	0.4426 (2)	0.0764 (8)	
H20	0.140257	0.567632	0.492089	0.092*	
C21	0.56890 (12)	0.34753 (10)	0.49988 (16)	0.0398 (4)	
H21	0.550585	0.341533	0.426391	0.048*	
C22	0.59092 (14)	0.39326 (13)	0.66186 (16)	0.0494 (5)	
H22	0.590355	0.425125	0.721445	0.059*	
C23	0.62978 (16)	0.32677 (14)	0.66178 (18)	0.0596 (6)	
H23	0.660522	0.304825	0.720784	0.072*	
C24	0.64896 (16)	0.22883 (12)	0.5175 (2)	0.0573 (6)	
H24A	0.615387	0.214479	0.451417	0.069*	
H24B	0.643667	0.190319	0.571681	0.069*	
C25	0.73985 (16)	0.23489 (12)	0.4922 (2)	0.0534 (5)	
C26	0.7648 (2)	0.28263 (17)	0.4128 (2)	0.0751 (8)	
H26	0.725232	0.313005	0.375349	0.090*	
C27	0.8488 (3)	0.2855 (2)	0.3887 (3)	0.1061 (13)	
H27	0.865192	0.317801	0.335160	0.127*	
C28	0.9074 (3)	0.2412 (3)	0.4429 (5)	0.1199 (16)	
H28	0.963483	0.243075	0.425907	0.144*	
C29	0.8836 (2)	0.1941 (3)	0.5221 (4)	0.1134 (14)	
H29	0.923590	0.164019	0.559236	0.136*	
C30	0.80019 (19)	0.19079 (17)	0.5476 (3)	0.0774 (8)	
H30	0.784610	0.158837	0.602104	0.093*	
O5	0.22185 (17)	0.5284 (2)	0.7504 (2)	0.1226 (10)	
H5A	0.267379	0.551864	0.766519	0.184*	
H5B	0.206889	0.512904	0.811700	0.184*	
O6	0.1289 (3)	0.4550 (2)	0.9369 (3)	0.1648 (15)	
N7	0.20211 (14)	0.50470 (13)	1.07952 (19)	0.0669 (6)	
C31	0.1313 (2)	0.4916 (2)	1.0252 (3)	0.0986 (11)	
H31	0.081506	0.509183	1.051085	0.118*	
C32	0.2811 (3)	0.4808 (3)	1.0442 (4)	0.1303 (17)	
H32A	0.271751	0.451665	0.978836	0.195*	
H32B	0.309898	0.451514	1.100831	0.195*	
H32C	0.314949	0.522959	1.029282	0.195*	
C33	0.2076 (3)	0.5490 (3)	1.1787 (3)	0.1280 (15)	
H33A	0.153125	0.569099	1.190220	0.192*	
H33B	0.247100	0.588469	1.171281	0.192*	
H33C	0.226384	0.518652	1.240114	0.192*	

### Atomic displacement parameters (Å^2^)

**Table:**

	*U*^11^	*U*^22^	*U*^33^	*U*^12^	*U*^13^	*U*^23^
Cu1	0.03603 (16)	0.02569 (14)	0.02567 (14)	−0.00356 (12)	−0.00096 (10)	−0.00254 (10)
Cu2	0.02823 (15)	0.02747 (15)	0.04268 (17)	−0.00080 (12)	−0.00426 (12)	0.00424 (12)
S1	0.0459 (3)	0.0306 (2)	0.02570 (19)	−0.00432 (19)	0.00091 (16)	−0.00219 (16)
O1	0.0489 (8)	0.0388 (7)	0.0286 (6)	−0.0049 (6)	−0.0020 (5)	0.0017 (5)
O2	0.0630 (9)	0.0373 (7)	0.0259 (6)	−0.0068 (6)	0.0003 (6)	−0.0048 (5)
O3	0.0747 (10)	0.0305 (7)	0.0370 (7)	−0.0090 (7)	−0.0045 (6)	0.0008 (5)
O4	0.0518 (9)	0.0589 (10)	0.0553 (9)	−0.0014 (8)	0.0141 (7)	−0.0097 (7)
N1	0.0442 (9)	0.0290 (7)	0.0308 (7)	−0.0054 (6)	0.0001 (6)	−0.0032 (6)
N2	0.0465 (9)	0.0310 (8)	0.0330 (8)	−0.0052 (7)	−0.0022 (6)	−0.0068 (6)
N3	0.0316 (8)	0.0333 (8)	0.0425 (8)	−0.0007 (6)	−0.0027 (6)	0.0010 (6)
N4	0.0295 (8)	0.0479 (9)	0.0482 (9)	−0.0023 (7)	−0.0026 (6)	0.0014 (7)
N5	0.0375 (8)	0.0316 (8)	0.0379 (8)	0.0011 (6)	−0.0037 (6)	0.0031 (6)
N6	0.0533 (10)	0.0346 (8)	0.0474 (9)	0.0085 (8)	0.0022 (7)	0.0087 (7)
C1	0.0544 (11)	0.0304 (9)	0.0307 (9)	−0.0062 (8)	0.0018 (8)	−0.0022 (7)
C2	0.0549 (12)	0.0320 (9)	0.0383 (10)	−0.0076 (9)	−0.0090 (8)	0.0025 (7)
C3	0.0626 (13)	0.0283 (9)	0.0452 (11)	−0.0068 (9)	−0.0114 (9)	0.0005 (8)
C4	0.0595 (13)	0.0415 (10)	0.0325 (9)	−0.0064 (9)	−0.0021 (8)	−0.0120 (8)
C5	0.0552 (12)	0.0354 (10)	0.0370 (10)	−0.0037 (9)	−0.0086 (8)	0.0009 (8)
C6	0.0745 (17)	0.0597 (14)	0.0563 (14)	−0.0149 (13)	−0.0090 (12)	−0.0150 (12)
C7	0.080 (2)	0.083 (2)	0.083 (2)	−0.0319 (18)	−0.0231 (16)	−0.0080 (17)
C8	0.0575 (16)	0.094 (2)	0.094 (2)	−0.0167 (16)	−0.0151 (16)	0.0204 (18)
C9	0.0572 (16)	0.086 (2)	0.0769 (18)	0.0080 (15)	−0.0006 (13)	0.0101 (15)
C10	0.0585 (14)	0.0544 (13)	0.0533 (13)	0.0036 (11)	−0.0082 (10)	−0.0055 (10)
C11	0.0338 (9)	0.0503 (12)	0.0404 (10)	−0.0012 (9)	−0.0030 (7)	−0.0024 (8)
C12	0.0384 (10)	0.0371 (10)	0.0542 (12)	0.0016 (8)	0.0018 (8)	−0.0109 (8)
C13	0.0376 (10)	0.0459 (12)	0.0644 (13)	−0.0046 (9)	−0.0037 (9)	−0.0163 (10)
C14	0.0331 (10)	0.0757 (16)	0.0555 (13)	−0.0050 (11)	0.0040 (9)	0.0079 (11)
C15	0.0326 (10)	0.0720 (16)	0.0493 (12)	0.0029 (10)	0.0054 (8)	−0.0023 (11)
C16	0.0492 (14)	0.094 (2)	0.0830 (19)	−0.0083 (14)	−0.0131 (13)	−0.0027 (16)
C17	0.0540 (17)	0.150 (4)	0.082 (2)	0.000 (2)	−0.0196 (14)	−0.007 (2)
C18	0.071 (2)	0.137 (4)	0.074 (2)	0.039 (2)	0.0059 (16)	0.010 (2)
C19	0.107 (3)	0.086 (2)	0.108 (3)	0.039 (2)	0.017 (2)	0.001 (2)
C20	0.0769 (19)	0.079 (2)	0.0731 (18)	0.0134 (16)	0.0012 (14)	−0.0134 (15)
C21	0.0464 (11)	0.0326 (9)	0.0390 (10)	0.0001 (8)	−0.0055 (8)	0.0051 (7)
C22	0.0575 (13)	0.0541 (13)	0.0356 (10)	0.0108 (10)	−0.0028 (9)	0.0034 (9)
C23	0.0733 (16)	0.0623 (15)	0.0419 (12)	0.0227 (12)	−0.0052 (10)	0.0143 (10)
C24	0.0644 (15)	0.0319 (10)	0.0764 (16)	0.0059 (10)	0.0105 (12)	0.0048 (10)
C25	0.0597 (14)	0.0374 (11)	0.0635 (14)	0.0041 (10)	0.0076 (11)	−0.0038 (10)
C26	0.082 (2)	0.0669 (18)	0.0775 (19)	−0.0057 (15)	0.0153 (15)	0.0040 (15)
C27	0.105 (3)	0.107 (3)	0.112 (3)	−0.034 (3)	0.047 (2)	−0.012 (2)
C28	0.070 (2)	0.127 (4)	0.167 (5)	−0.008 (2)	0.031 (3)	−0.035 (3)
C29	0.062 (2)	0.112 (3)	0.163 (4)	0.018 (2)	−0.016 (2)	−0.018 (3)
C30	0.0710 (19)	0.0675 (18)	0.092 (2)	0.0128 (15)	−0.0051 (15)	0.0025 (15)
O5	0.0773 (16)	0.200 (3)	0.0881 (17)	−0.0242 (18)	−0.0116 (13)	−0.028 (2)
O6	0.211 (4)	0.168 (3)	0.105 (2)	−0.027 (3)	−0.061 (2)	−0.021 (2)
N7	0.0562 (13)	0.0857 (17)	0.0590 (13)	0.0047 (11)	0.0061 (10)	0.0064 (11)
C31	0.080 (2)	0.117 (3)	0.095 (3)	−0.005 (2)	−0.0134 (19)	0.013 (2)
C32	0.089 (3)	0.170 (4)	0.135 (4)	0.038 (3)	0.036 (3)	0.036 (3)
C33	0.133 (4)	0.161 (4)	0.088 (3)	−0.021 (3)	−0.004 (2)	−0.021 (3)

### Geometric parameters (Å, º)

**Table:**

Cu1—O1^i^	1.9564 (12)	C12—C13	1.354 (3)
Cu1—O1	1.9564 (12)	C13—H13	0.9300
Cu1—N1^i^	1.9951 (14)	C14—H14A	0.9700
Cu1—N1	1.9951 (14)	C14—H14B	0.9700
Cu2—O2	2.4912 (12)	C14—C15	1.498 (3)
Cu2—N3^ii^	2.0210 (15)	C15—C16	1.375 (3)
Cu2—N3	2.0210 (15)	C15—C20	1.359 (4)
Cu2—N5	2.0103 (15)	C16—H16	0.9300
Cu2—N5^ii^	2.0103 (15)	C16—C17	1.377 (4)
S1—O1	1.5139 (13)	C17—H17	0.9300
S1—O2	1.4653 (13)	C17—C18	1.345 (6)
S1—O3	1.4608 (14)	C18—H18	0.9300
S1—O4	1.4567 (15)	C18—C19	1.354 (5)
N1—C1	1.326 (2)	C19—H19	0.9300
N1—C2	1.375 (2)	C19—C20	1.398 (4)
N2—C1	1.336 (2)	C20—H20	0.9300
N2—C3	1.367 (2)	C21—H21	0.9300
N2—C4	1.463 (2)	C22—H22	0.9300
N3—C11	1.320 (2)	C22—C23	1.354 (3)
N3—C12	1.369 (2)	C23—H23	0.9300
N4—C11	1.341 (2)	C24—H24A	0.9700
N4—C13	1.364 (3)	C24—H24B	0.9700
N4—C14	1.471 (3)	C24—C25	1.503 (3)
N5—C21	1.316 (2)	C25—C26	1.380 (4)
N5—C22	1.375 (2)	C25—C30	1.386 (4)
N6—C21	1.341 (2)	C26—H26	0.9300
N6—C23	1.363 (3)	C26—C27	1.389 (4)
N6—C24	1.467 (3)	C27—H27	0.9300
C1—H1	0.9300	C27—C28	1.363 (6)
C2—H2	0.9300	C28—H28	0.9300
C2—C3	1.347 (3)	C28—C29	1.366 (6)
C3—H3	0.9300	C29—H29	0.9300
C4—H4A	0.9700	C29—C30	1.384 (5)
C4—H4B	0.9700	C30—H30	0.9300
C4—C5	1.502 (3)	O5—H5A	0.8500
C5—C6	1.390 (3)	O5—H5B	0.8499
C5—C10	1.377 (3)	O6—C31	1.266 (5)
C6—H6	0.9300	N7—C31	1.285 (4)
C6—C7	1.382 (4)	N7—C32	1.425 (4)
C7—H7	0.9300	N7—C33	1.451 (4)
C7—C8	1.372 (5)	C31—H31	0.9300
C8—H8	0.9300	C32—H32A	0.9600
C8—C9	1.360 (4)	C32—H32B	0.9600
C9—H9	0.9300	C32—H32C	0.9600
C9—C10	1.383 (4)	C33—H33A	0.9600
C10—H10	0.9300	C33—H33B	0.9600
C11—H11	0.9300	C33—H33C	0.9600
C12—H12	0.9300		
			
O1^i^—Cu1—O1	180.0	C13—C12—H12	125.2
O1—Cu1—N1^i^	89.25 (6)	N4—C13—H13	126.9
O1—Cu1—N1	90.75 (6)	C12—C13—N4	106.24 (17)
O1^i^—Cu1—N1^i^	90.75 (6)	C12—C13—H13	126.9
O1^i^—Cu1—N1	89.25 (6)	N4—C14—H14A	109.3
N1^i^—Cu1—N1	180.0	N4—C14—H14B	109.3
N3—Cu2—O2	86.02 (5)	N4—C14—C15	111.54 (18)
N3^ii^—Cu2—O2	93.98 (5)	H14A—C14—H14B	108.0
N3—Cu2—N3^ii^	180.0	C15—C14—H14A	109.3
N5—Cu2—O2	93.63 (5)	C15—C14—H14B	109.3
N5^ii^—Cu2—O2	86.36 (5)	C16—C15—C14	121.5 (3)
N5—Cu2—N3^ii^	88.00 (6)	C20—C15—C14	120.4 (2)
N5—Cu2—N3	92.00 (6)	C20—C15—C16	118.1 (3)
N5^ii^—Cu2—N3	87.99 (6)	C15—C16—H16	119.2
N5^ii^—Cu2—N3^ii^	92.00 (6)	C15—C16—C17	121.7 (3)
N5^ii^—Cu2—N5	180.00 (4)	C17—C16—H16	119.2
O2—S1—O1	106.99 (8)	C16—C17—H17	120.5
O3—S1—O1	108.69 (8)	C18—C17—C16	119.0 (3)
O3—S1—O2	110.68 (8)	C18—C17—H17	120.5
O4—S1—O1	106.63 (9)	C17—C18—H18	119.3
O4—S1—O2	111.57 (9)	C17—C18—C19	121.3 (3)
O4—S1—O3	112.02 (10)	C19—C18—H18	119.3
S1—O1—Cu1	121.58 (8)	C18—C19—H19	120.3
S1—O2—Cu2	151.93 (9)	C18—C19—C20	119.3 (4)
C1—N1—Cu1	127.18 (13)	C20—C19—H19	120.3
C1—N1—C2	105.82 (15)	C15—C20—C19	120.5 (3)
C2—N1—Cu1	127.00 (12)	C15—C20—H20	119.7
C1—N2—C3	107.53 (15)	C19—C20—H20	119.7
C1—N2—C4	126.30 (16)	N5—C21—N6	111.39 (17)
C3—N2—C4	126.14 (16)	N5—C21—H21	124.3
C11—N3—Cu2	125.23 (13)	N6—C21—H21	124.3
C11—N3—C12	105.81 (16)	N5—C22—H22	125.7
C12—N3—Cu2	128.84 (13)	C23—C22—N5	108.54 (19)
C11—N4—C13	107.44 (16)	C23—C22—H22	125.7
C11—N4—C14	125.85 (18)	N6—C23—H23	126.4
C13—N4—C14	126.58 (17)	C22—C23—N6	107.22 (18)
C21—N5—Cu2	125.28 (12)	C22—C23—H23	126.4
C21—N5—C22	106.07 (16)	N6—C24—H24A	109.0
C22—N5—Cu2	127.77 (14)	N6—C24—H24B	109.0
C21—N6—C23	106.77 (17)	N6—C24—C25	112.82 (19)
C21—N6—C24	125.82 (18)	H24A—C24—H24B	107.8
C23—N6—C24	127.27 (18)	C25—C24—H24A	109.0
N1—C1—N2	110.80 (16)	C25—C24—H24B	109.0
N1—C1—H1	124.6	C26—C25—C24	121.4 (2)
N2—C1—H1	124.6	C26—C25—C30	118.7 (3)
N1—C2—H2	125.4	C30—C25—C24	119.9 (2)
C3—C2—N1	109.25 (16)	C25—C26—H26	119.9
C3—C2—H2	125.4	C25—C26—C27	120.2 (3)
N2—C3—H3	126.7	C27—C26—H26	119.9
C2—C3—N2	106.60 (17)	C26—C27—H27	119.7
C2—C3—H3	126.7	C28—C27—C26	120.5 (4)
N2—C4—H4A	109.0	C28—C27—H27	119.7
N2—C4—H4B	109.0	C27—C28—H28	120.1
N2—C4—C5	112.99 (16)	C27—C28—C29	119.8 (4)
H4A—C4—H4B	107.8	C29—C28—H28	120.1
C5—C4—H4A	109.0	C28—C29—H29	119.8
C5—C4—H4B	109.0	C28—C29—C30	120.5 (4)
C6—C5—C4	118.9 (2)	C30—C29—H29	119.8
C10—C5—C4	123.13 (18)	C25—C30—H30	119.9
C10—C5—C6	118.0 (2)	C29—C30—C25	120.3 (3)
C5—C6—H6	119.7	C29—C30—H30	119.9
C7—C6—C5	120.5 (3)	H5A—O5—H5B	104.5
C7—C6—H6	119.7	C31—N7—C32	123.0 (4)
C6—C7—H7	119.9	C31—N7—C33	122.0 (3)
C8—C7—C6	120.2 (3)	C32—N7—C33	114.9 (3)
C8—C7—H7	119.9	O6—C31—N7	120.5 (4)
C7—C8—H8	120.0	O6—C31—H31	119.7
C9—C8—C7	120.0 (3)	N7—C31—H31	119.7
C9—C8—H8	120.0	N7—C32—H32A	109.5
C8—C9—H9	120.0	N7—C32—H32B	109.5
C8—C9—C10	120.0 (3)	N7—C32—H32C	109.5
C10—C9—H9	120.0	H32A—C32—H32B	109.5
C5—C10—C9	121.3 (2)	H32A—C32—H32C	109.5
C5—C10—H10	119.3	H32B—C32—H32C	109.5
C9—C10—H10	119.3	N7—C33—H33A	109.5
N3—C11—N4	110.99 (18)	N7—C33—H33B	109.5
N3—C11—H11	124.5	N7—C33—H33C	109.5
N4—C11—H11	124.5	H33A—C33—H33B	109.5
N3—C12—H12	125.2	H33A—C33—H33C	109.5
C13—C12—N3	109.52 (18)	H33B—C33—H33C	109.5
			
Cu1—N1—C1—N2	179.36 (13)	C10—C5—C6—C7	0.2 (4)
Cu1—N1—C2—C3	−179.47 (15)	C11—N3—C12—C13	−0.3 (2)
Cu2—N3—C11—N4	177.06 (12)	C11—N4—C13—C12	0.6 (2)
Cu2—N3—C12—C13	−176.47 (14)	C11—N4—C14—C15	108.7 (2)
Cu2—N5—C21—N6	170.35 (13)	C12—N3—C11—N4	0.7 (2)
Cu2—N5—C22—C23	−169.85 (16)	C13—N4—C11—N3	−0.9 (2)
O1—S1—O2—Cu2	−74.37 (19)	C13—N4—C14—C15	−66.4 (3)
O2—S1—O1—Cu1	−121.26 (9)	C14—N4—C11—N3	−176.80 (18)
O3—S1—O1—Cu1	119.19 (9)	C14—N4—C13—C12	176.5 (2)
O3—S1—O2—Cu2	43.9 (2)	C14—C15—C16—C17	−179.5 (3)
O4—S1—O1—Cu1	−1.75 (11)	C14—C15—C20—C19	−179.8 (3)
O4—S1—O2—Cu2	169.36 (16)	C15—C16—C17—C18	−0.7 (5)
N1—C2—C3—N2	−0.2 (2)	C16—C15—C20—C19	0.3 (4)
N2—C4—C5—C6	−160.3 (2)	C16—C17—C18—C19	0.2 (5)
N2—C4—C5—C10	20.2 (3)	C17—C18—C19—C20	0.5 (5)
N3—C12—C13—N4	−0.2 (2)	C18—C19—C20—C15	−0.7 (5)
N4—C14—C15—C16	99.3 (3)	C20—C15—C16—C17	0.5 (4)
N4—C14—C15—C20	−80.7 (3)	C21—N5—C22—C23	−0.2 (3)
N5—C22—C23—N6	0.0 (3)	C21—N6—C23—C22	0.2 (3)
N6—C24—C25—C26	−62.5 (3)	C21—N6—C24—C25	98.7 (3)
N6—C24—C25—C30	118.9 (3)	C22—N5—C21—N6	0.4 (2)
C1—N1—C2—C3	0.4 (2)	C23—N6—C21—N5	−0.4 (2)
C1—N2—C3—C2	−0.2 (2)	C23—N6—C24—C25	−76.5 (3)
C1—N2—C4—C5	−108.7 (2)	C24—N6—C21—N5	−176.43 (19)
C2—N1—C1—N2	−0.5 (2)	C24—N6—C23—C22	176.2 (2)
C3—N2—C1—N1	0.4 (2)	C24—C25—C26—C27	−178.0 (3)
C3—N2—C4—C5	68.9 (3)	C24—C25—C30—C29	177.7 (3)
C4—N2—C1—N1	178.41 (17)	C25—C26—C27—C28	0.1 (6)
C4—N2—C3—C2	−178.14 (19)	C26—C25—C30—C29	−1.0 (4)
C4—C5—C6—C7	−179.3 (2)	C26—C27—C28—C29	−0.5 (7)
C4—C5—C10—C9	180.0 (2)	C27—C28—C29—C30	0.2 (7)
C5—C6—C7—C8	−0.7 (5)	C28—C29—C30—C25	0.5 (6)
C6—C5—C10—C9	0.4 (4)	C30—C25—C26—C27	0.7 (4)
C6—C7—C8—C9	0.6 (5)	C32—N7—C31—O6	1.1 (6)
C7—C8—C9—C10	0.0 (5)	C33—N7—C31—O6	177.5 (4)
C8—C9—C10—C5	−0.6 (4)		

Symmetry codes: (i) −*x*+1, −*y*+1, −*z*+2; (ii) −*x*+1, −*y*+1, −*z*+1.

### Hydrogen-bond geometry (Å, º)

*Cg* is the centroid of the C5–C10 ring.

**Table:**

*D*—H···*A*	*D*—H	H···*A*	*D*···*A*	*D*—H···*A*
O5—H5*A*···O4	0.85	1.96	2.772 (3)	159
O5—H5*B*···O6	0.85	2.29	3.106 (5)	160
C1—H1···O2	0.93	2.23	3.131 (2)	164
C2—H2···O1^i^	0.93	2.60	2.966 (2)	104
C2—H2···O3^i^	0.93	2.34	3.227 (2)	159
C4—H4*A*···O3^iii^	0.97	2.55	3.445 (3)	153
C10—H10···N2	0.93	2.54	2.866 (3)	101
C11—H11···O2	0.93	2.40	2.990 (2)	121
C12—H12···O3^ii^	0.93	2.48	3.378 (2)	162
C14—H14*A*···O5	0.97	2.50	3.425 (4)	159
C21—H21···O2^ii^	0.93	2.57	3.038 (2)	112
C21—H21···O3^ii^	0.93	2.36	3.274 (2)	170
C24—H24*B*···O4^iii^	0.97	2.40	3.346 (3)	164
C32—H32*A*···O6	0.96	2.29	2.705 (7)	105
C13—H13···*Cg*4^iv^	0.93	2.87	3.759 (3)	161
C14—H14*B*···*Cg*4^iv^	0.97	2.99	3.711 (3)	132

Symmetry codes: (i) −*x*+1, −*y*+1, −*z*+2; (ii) −*x*+1, −*y*+1, −*z*+1; (iii) −*x*+1, *y*−1/2, −*z*+3/2; (iv) *x*, −*y*+1/2, *z*−1/2.
