# Supplementary material for: Crystal structure of catena-poly[[[bis­(1-benzyl­imidazole-κN)copper(II)]-μ-sulfato-κ2 O:O′-[tetra­kis­(1-benzyl­imidazole-κN)copper(II)]-μ-sulfato-κ2 O:O′] N,N-di­methyl­formamide disolvate dihydrate]
Source: Acta Crystallogr E Crystallogr Commun. 2022 Sep 6;78(Pt 10):974–8. doi: 10.1107/S2056989022008714 (PMC9535829; doi:10.1107/S2056989022008714)
Supplement: Supplementary file 3 [file e-78-00974-sup3.pdf]

Supporting Information

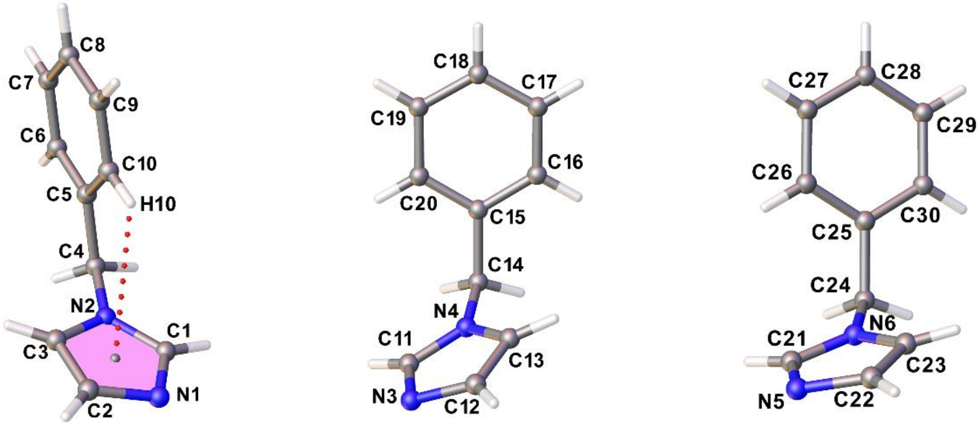

Figure S1. Conformations of bzi molecules in the title compound.

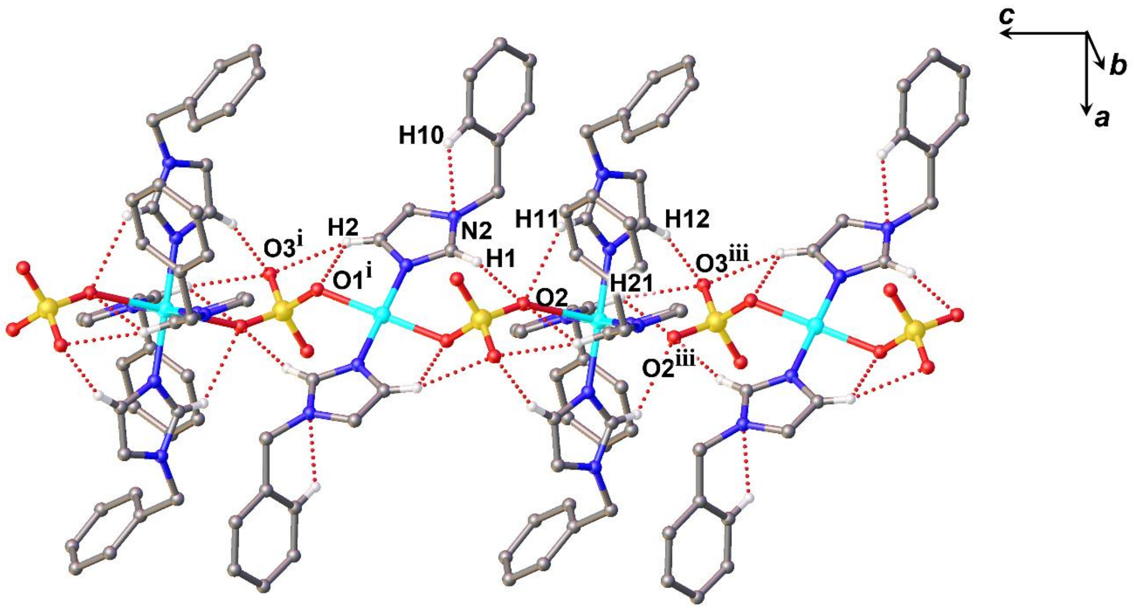

Figure S2. Intramolecular hydrogen bonding interactions in the 1D chain of the title compound. Some hydrogen atoms located at carbon atoms and lattice water and DMF solvate molecules are omitted for clarity. (symmetry codes (i) =-x+1, -y+1, -z+2 and (iii) = -x+1, -y+1, -z+1)

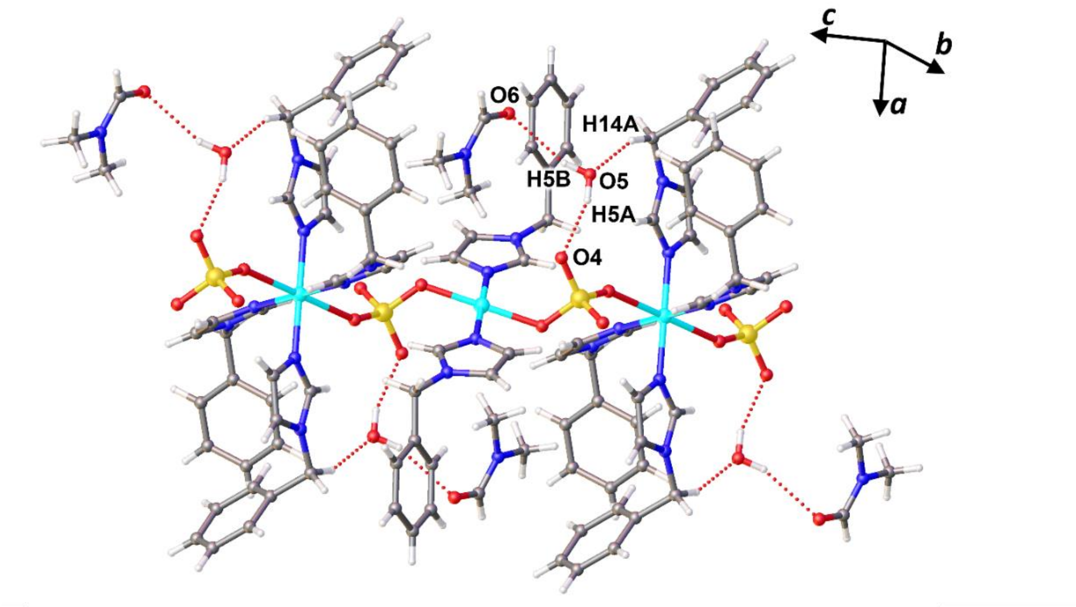

Figure S3. Intermolecular hydrogen bonding interactions between 1D chains and lattice solvent molecules of the title compound.

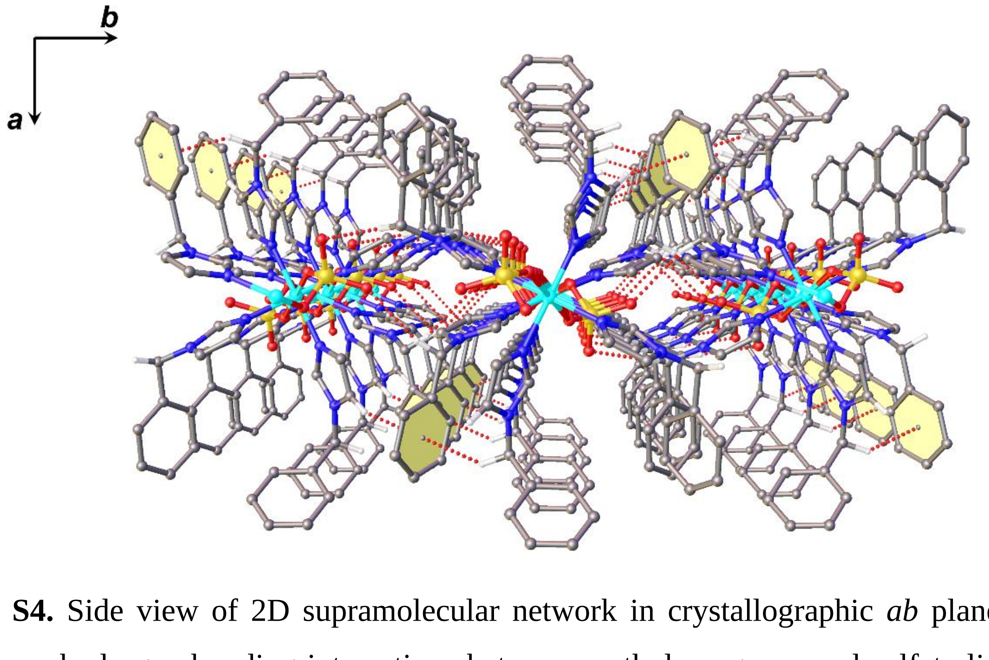

Figure S4. Side view of 2D supramolecular network in crystallographic *ab* plane through hydrogen bonding interactions between methylene group and sulfate ligand, and the C-H···π interactions.

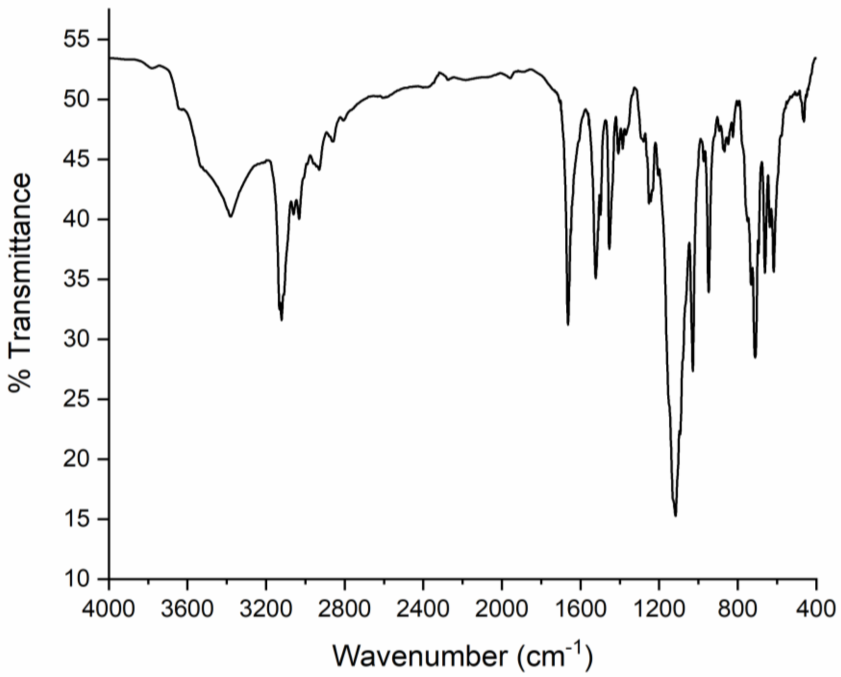

Figure S5. IR spectrum of the title compound

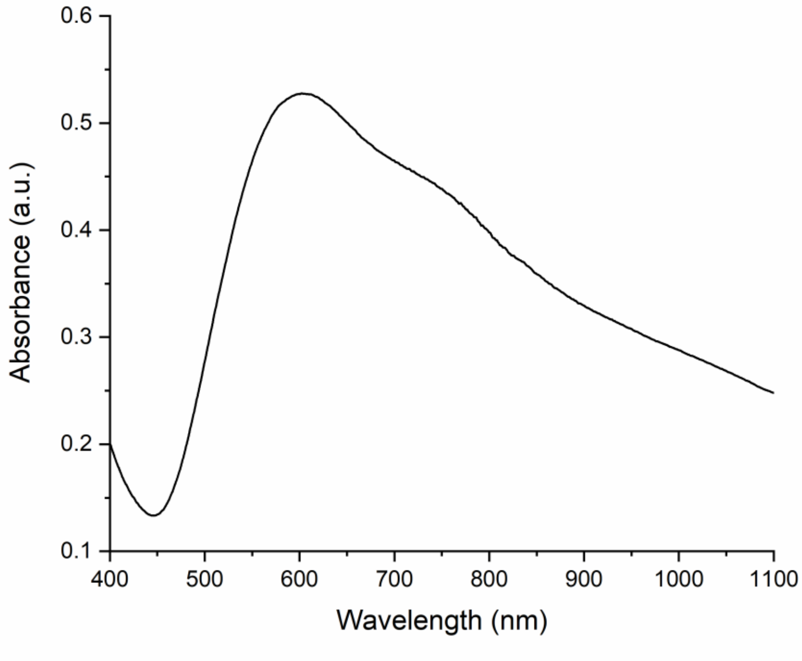

Figure S6. Solid state diffuse reflectance spectrum of the title compound

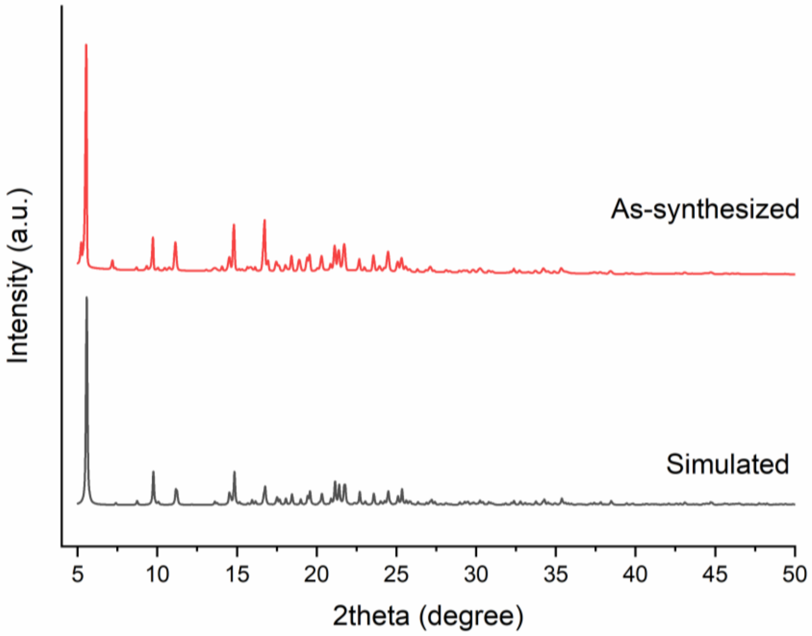

Figure S7. PXRD patterns of the title compound

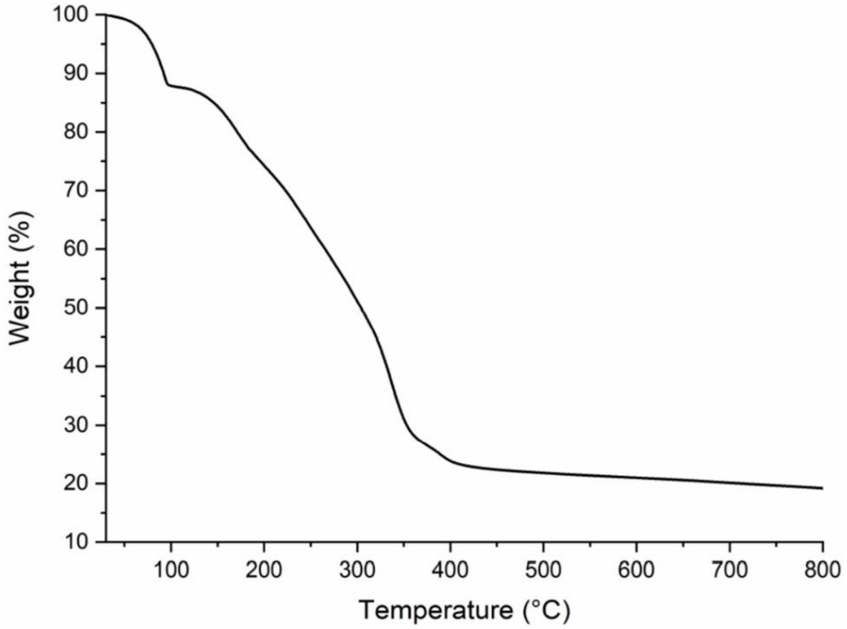

Figure S8. TGA curve of the title compound
